# A Processing Pipeline for Quantifying Lenticulostriate Artery Vascular Volume in Subcortical Nuclei

**DOI:** 10.3389/fneur.2021.700476

**Published:** 2021-08-16

**Authors:** Ning Wei, Xianchang Zhang, Jing An, Yan Zhuo, Zihao Zhang

**Affiliations:** ^1^China National Clinical Research Center for Neurological Diseases, Beijing Tiantan Hospital, Capital Medical University, Beijing, China; ^2^MR Collaboration, Siemens Healthcare Ltd., Beijing, China; ^3^Siemens Shenzhen Magnetic Resonance Ltd., Shenzhen, China; ^4^State Key Laboratory of Brain and Cognitive Science, Institute of Biophysics, Chinese Academy of Sciences, Beijing, China; ^5^University of Chinese Academy of Sciences, Beijing, China

**Keywords:** TOF-MRA, lenticulostriate artery, subcortical nuclei, 7T, vascular volume

## Abstract

Lenticulostriate arteries (LSAs) supply blood to the basal ganglia region. Its lesion causes lacunar stroke and resulting neurological syndromes. However, due to its small caliber and large individual variance, the evaluation of LSAs was limited to descriptive and objective measurements. In this study, we aimed to develop a post-processing method to quantify LSAs in subcortical regions and compare their vascular volume to conventional LSA measurements. A processing pipeline was designed to extract subcortical areas in individual spaces while screening out vessels. The vascular volume of LSAs in the subcortical region was calculated from time-of-flight-magnetic resonance angiography (TOF-MRA) at 7 Tesla. The reproducibility was tested to be good for the vascular volume (*n* = 5, ICC_A_ = 0.84). Comparing the results to conventional measurements, the vascular volume was significantly correlated with the number of branches (*r* = 0.402, *p* < 0.001) and the length (*r* = 0.246, *p* = 0.032) of LSAs. By applying the method to a group of healthy volunteers (*n* = 40), we found that most LSAs crossing through the putamen which thereby has the highest vascular density among subcortical nuclei. In general, we proposed a semi-automated processing pipeline for quantifying the vascular volume of LSAs in subcortical regions. The novel method was tested to be robust and provided reasonable results. This method revealed spatial relationships among the perforating arteries and basal ganglia. The vascular volume can be used to evaluated blood supply of subcortical regions, benefiting the radiologic evaluation of neurodegenerative diseases caused by small vascular lesions.

## Introduction

Lenticulostriate arteries (LSAs) represent the major microvasculature of the middle cerebral arteries and supply blood to the basal ganglia and internal capsules (1, 2). The occlusion of LSAs will cause lacunar infarcts in subcortical structures, leading to motor deficits, sensory deficits, and cognitive dysfunction (3). Imaging LSAs could be useful for clinical applications and provide insights into the mechanisms underlying cerebral microvascular disease development. Digital subtraction angiography (DSA) has been the gold standard for intracranial vascular imaging (4), but this method requires intravenous contrast and is not suitable for routine evaluations. With advances in imaging techniques, magnetic resonance angiography (MRA) has been widely used in clinical medicine because of its non-invasiveness. In the past decade, time-of-flight magnetic resonance angiography (TOF-MRA) at 7 Tesla (7T) has been shown to produce markedly superior blood-to-tissue contrast, which has enabled the non-invasive visualization of human brain microvasculature, *in vivo*, especially the microvasculature of LSAs (5, 6). Lenticulostriate artery angiography is useful as a diagnostic tool in clinical and research settings for detecting diseases, such as small vessel disease, lacunar infarcts, and vascular dementia. Recent studies have found LSA abnormalities in patients with hypertension (7), lacunar infarcts (8), and vascular dementia (9).

The distribution and morphological characteristics of cerebral small vessels play important roles in diagnosis and treatment of various cerebrovascular diseases (10, 11). However, the analysis tools of LSAs, particularly the quantitative characterization, are still limited. In most studies, the number of LSA stems and branches arising from the first segment of the bilateral ACAs and MCAs are counted manually (7, 9, 12). Some studies have measured the angles, lengths, and curvatures of the longest LSAs on 2D maximum intensity projection (MIP) images (6, 13, 14) or 3D extracted vessels (15, 16). In addition, LSA orifices (8) and vascular densities obtained with rough threshold segmentations (17) have also been proposed. However, due to morphologic variations in LSA structures, these quantification methods have poor sensitivity and stability in being able to evaluate patient groups and sometimes have led to controversial clinical study results (12, 18).

Based on LSA spatial relationships and those of its supplying territories, our study introduces a novel method to quantify LSA vascular volume in subcortical areas to estimate basal ganglia and internal capsule blood supplies. A semi-automatic post-processing pipeline was designed and adjusted to produce LSA vascular volume, objectively. Conventional quantitative indicators, such as the number of branches and lengths of the LSAs were compared with LSA vascular volume.

## Materials and Methods

### Participants

A total of 45 participants were enrolled in this study, separated into validation group (G_val_) and application group (G_app_). G_val_ was used to validate the method and assess its robustness and reproducibility. It included five healthy participants (Aged 25 ± 2.3 years, 3 males). These participants underwent MRI acquisition twice on the same day, with repositioning between two scans. G_app_ was applied to find the prevalence of LSAs in basal ganglia region and formed by 40 healthy volunteers (Aged 40.7 ± 9.7 years, 21 males). Informed consent was obtained from all participants and was approved by the local institutional review board.

### Magnetic Resonance Imaging

All MRI scans were performed on a 7T whole-body research MR scanner (Siemens Healthcare, Erlangen, Germany). A birdcage transmission and a 32-channel receiving head coil (Nova Medical, MA, USA) were used for all scanning procedures. T1-weighted magnetization-prepared rapid gradient echo (T1w MPRAGE) and TOF-MRA were collected for every participant.

Two protocols of TOF-MRA sequence were optimized to visualize LSAs. To evaluate the reproducibility of our method, whole-brain TOF-MRA with isotropic 0.40 mm resolution was acquired in G_val_ with the following parameters: field of view (FOV) = 205 × 175 × 107 mm^3^, number of slabs = 6, repetition time (TR) = 18 ms, echo time (TE) = 4.03 ms, flip angle (FA) = 22°, bandwidth (BW) = 158 Hz/Px, generalized auto-calibrating partial parallel acquisition (GRAPPA) factor = 3, time of acquisition (TA) = 9 min 28 s. For the G_app_, single-slab TOF-MRA was optimized to shorten the acquisition time (9). The imaging slab was positioned obliquely axial covering the bottom of the MCA and basal ganglia. Detailed parameters were FOV = 180 × 135 × 52 mm^3^, resolution = 0.23 × 0.23 × 0.36 mm^3^, TR = 15ms, TE = 3.57ms, FA = 20°, BW = 151 Hz/Px, GRAPPA factor = 2, TA = 8 min 20 s. The T1w MPRAGE was obtained for structural images in both groups, with the following parameters: FOV = 224 × 224 × 179 mm^3^, resolution = 0.70 × 0.70 × 0.70 mm^3^, TR = 3,000ms, TE = 3.23 ms, inversion time = 1,050ms, FA = 8°, BW = 320 Hz/Px, GRAPPA factor = 3, TA = 5 min 54 s.

### Image Analysis

#### Pre-processing

**Figure 1:**
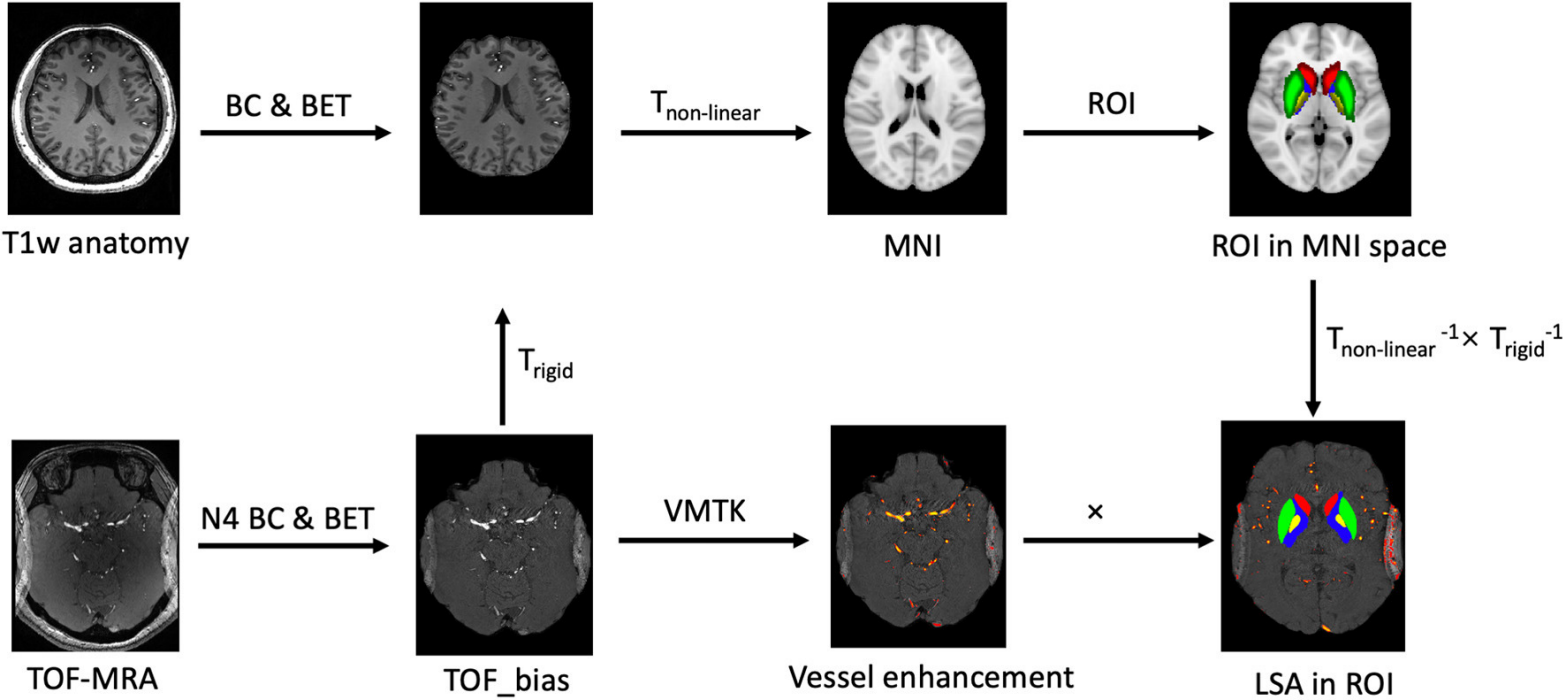
The workflow for calculating lenticulostriate artery (LSA) vascular volume. BC, bias correction; BET, brain extraction tool using FSL software; VMTK, Vascular Modeling Toolkit.

The pre-processing was performed in FSL software, a comprehensive library of analytic tools (19). The processing workflow is shown in Figure 1. First, the T1w anatomical dataset was registered to the Montreal Neurological Institute (MNI) brain atlas. Non-linear registrations (FNIRT) were used following affine linear transformations (FLIRT) for maximal accuracy. Second, we chose the regions of interest (ROI) indicating the LSA supply territories to build a mask in the MNI space. The selected areas included the putamen, globus pallidus, and caudate nuclei from the Harvard-Oxford Subcortical Structural Atlas and the posterior limbs of the internal capsule from the JHU ICBM-DTI-81 White-Matter labels. On the other hand, the TOF-MRA dataset was aligned to its corresponding T1w anatomical dataset. The N4 bias correction was applied to resolve signal inhomogeneity caused by the dielectric effects of ultra-high fields (20). Finally, rigid and non-linear transformations were concatenated to transform the ROI masks into individual TOF-MRA spaces.

#### Vessel Enhancement and Filtering

To enhance vessel-to-tissue contrast and suppress non-vascular structures and image noise, we used vessel enhancement filters from the Vascular Modeling Toolkit (21). One voxel of LSA was selected as a seed point. Based on the morphology of LSAs and spatial resolutions of TOF-MRA, vessel enhancement filter parameters were optimized as: minimum vessel diameter = 1 voxel, maximum vessel diameter = 5 voxels, vessel contrast = 100, suppress plates = 10%, and suppress blobs = 40%. Then, we used the previously generated ROI masks to extract enhanced vasculatures to calculate the volume of LSAs, as shown in Figure 1.

Blood vessel volume was analyzed with the MATLAB 2016 software program. We applied a volume ratio scheme to find an appropriate threshold for screening out complete vessels while filtering out noise from the parenchymal tissues. The thresholds were adjusted until vessel-to-tissue volume ratios changed >0.2%. The vascular volume was defined as the number of voxels above the threshold multiplied by the imaging resolution.

#### Quantifying the Morphologic Characteristics of Lenticulostriate Arteries

Three dimensional (3D) MRA image reconstructions and analyses were performed using a free and open-source code software from Horos (https://horosproject.org). We counted the number of LSA stems and branches arising from the first segment of the bilateral ACAs (A1) and MCAs (M1). Only blood vessels of the LSA-supplying territories (the previously mentioned ROIs) were counted. Stems were defined as LSAs directly connected to A1s or M1s. Branches were defined as daughter vessels arising from parent LSA stems without any subordinate branches. If a trunk had no branches, it was recorded as both stem and branch. To measure the maximal length of the LSAs, MIP was reconstructed in the coronal view with a slab thickness of 28mm covering the LSAs and MCAs. The maximal length was determined as the distance from MCA to the visible end of the longest LSA.

### Statistical Analyses

The inter-class correlation coefficient (ICC) was used as the measure for reproducibility, assessing the absolute agreement (ICC_A_) between both scans of G_val_. In addition, the data of G_app_ was used for quantitative analysis. All the quantitative data were presented as means ± standard deviations (SDs). The level of significance was set at *p* < 0.05. The volume of LSAs calculated with our new method was compared with conventional LSA measurements using Pearson's correlation coefficient. All statistical analyses were carried out using SPSS vision 21 software.

## Results

**Figure 2:**
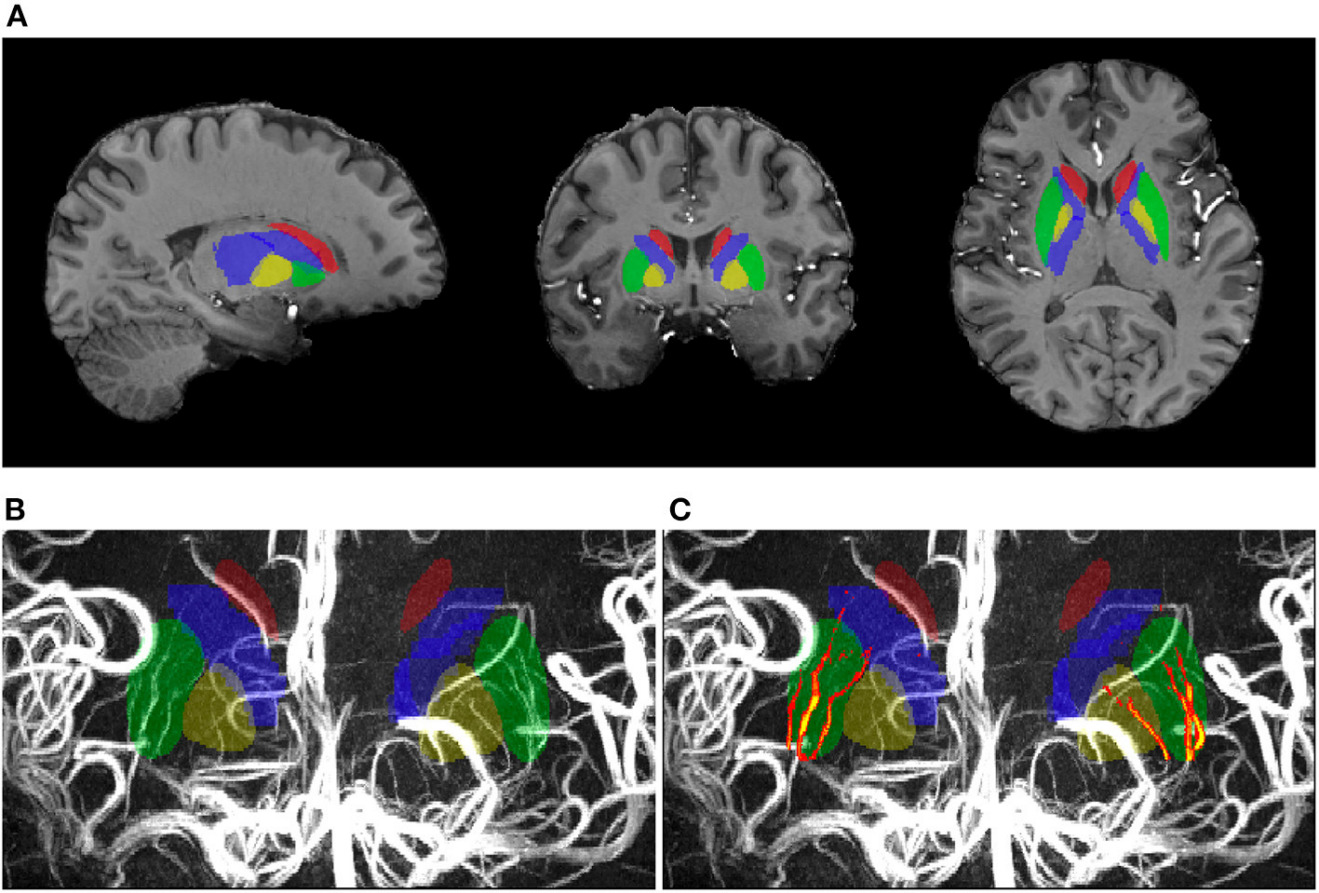
The subcortical regions of interest (ROIs) and LSA vascular volume in one participant. **(A)** The ROIs in the anatomical image; **(B,C)** The vascular voxels identified in the ROIs concide well with the vasculature in the raw image.

**Figure 3:**
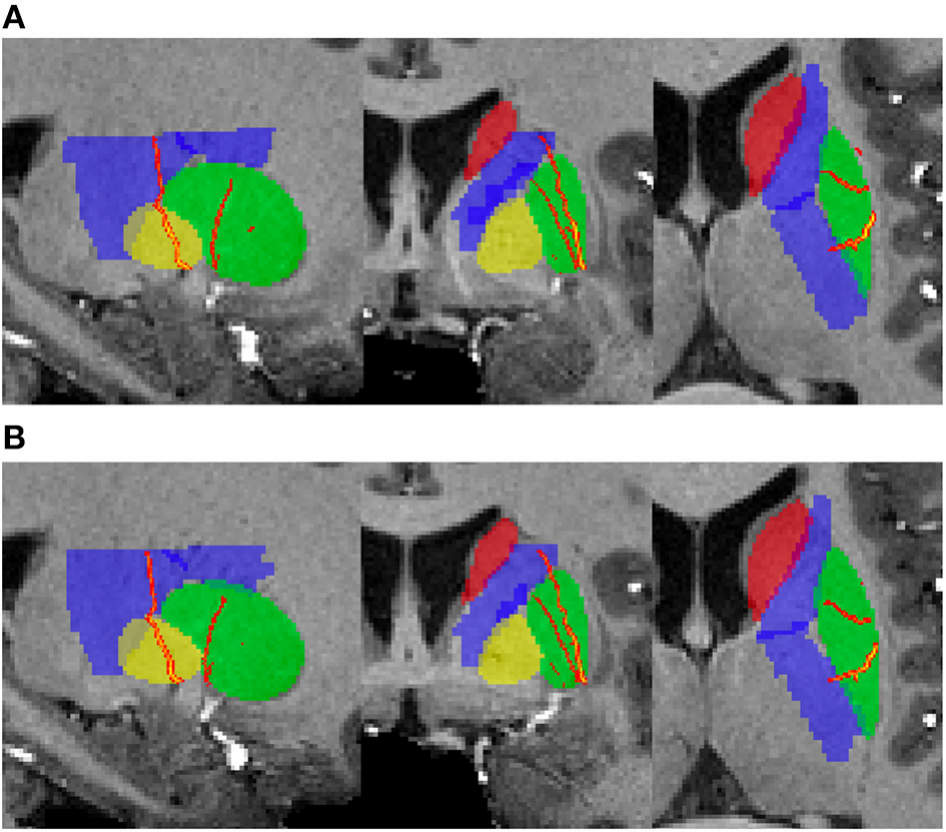
The extracted LSA vasculatures of one hemisphere in the reproducibility testing. **(A)** The first scan; **(B)** The second scan.

Figure 2 shows an example of subcortical ROIs and LSA voxels. The subcortical nuclei and basal ganglia were well-segmented according to the anatomic images. The LSA vasculature was clearly demonstrated after vessel enhancement and filtering. The volumes of subcortical ROIs have an ICC_A_ = 0.98 (95% CI = 0.88–0.99), confirming excellent registration for ROI definition. The inter-scan reproducibility of quantifying LSA volume was good with an ICC_A_ = 0.84 (95% CI = 0.47–0.96). An example of reproducibility test was exhibited in Figure 3. The discrepancies between the repeated scans occurred mostly for tiny branches of LSA which was sensitive to partial volume effect.

**Figure 4:**
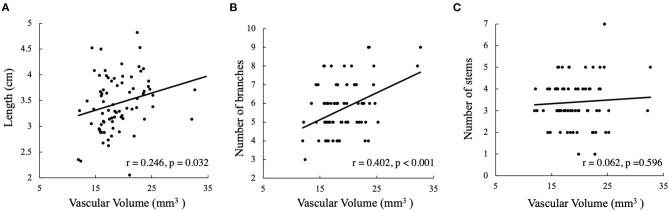
Correlation analyses of lenticulostriate artery (LSA) volume and conventional measurements; LSA vascular volume vs. **(A)** LSA lengths, **(B)** the number of LSA branches, and **(C)** the number of LSA stems.

Two participants in G_app_ were excluded from the analyses because voxels of large arteries were involved in the basilar ROI and contaminated the results. In total, 38 participants (76 hemispheres) were used for the statistical analyses. The LSA voxel numbers in the subcortical nuclei and internal capsules were 998.01 ± 206.88, which corresponded to a volume of 19.01 ± 0.45 mm^3^. There were 5.71 ± 1.41 LSA branches and 3.39 ± 1.07 stems in each hemisphere, and the length of the longest branch was 3.44 ± 0.53 cm. The vascular volume was significantly correlated with the number of LSA branches (*r* = 0.402, *p* < 0.001) and the LSA lengths (*r* = 0.246, *p* = 0.032), but no correlation was found between the vascular volume and the number of LSA stems (*r* = 0.062, *p* = 0.596), as shown in Figure 4. The LSA voxel numbers in different subcortical regions (putamen, globus pallidus, caudate nucleus, and internal capsule) were further analyzed. We found that 90% of the visible perforating branches funneled through the putamen, which can be observed in Figure 2.

## Discussion

Our study proposed a novel method for quantifying perforating arteries in subcortical regions. LSA vascular volume, equivalent to voxel numbers, reflected regions of subcortical blood supply. In previous studies, stems, branches, and lengths were used to describe LSA morphologies. Compared with these traditional measurements, the vascular volume in subcortical ROIs was significantly correlated with the number of LSA branches and the LSA lengths. Conversely, LSA vascular volume was not correlated with the number of LSA stems, which could be explained by larger perforating arteries dividing into numerous branches before entering the brain substance (22, 23). Lenticulostriate artery volume assessments were able to estimate blood supplies in the subcortical regions more appropriately.

This new analytic method has two main advantages over traditional methods. First, the vascular volume is comprehensive representations of LSA numbers, lengths, and diameters. In a previous study, negative correlations were reported between LSA numbers and diameters (the higher the number of branches, the smaller the branch diameters, and vice versa) (24). Vascular volume measurements provided more comprehensive LSA quantifications in the subcortical regions, which was likely related to the blood perfusion in this area. In addition, the analytic methods represent an automatic post-processing pipeline that avoids the subjective bias of traditional measurements. In previous studies, investigators had to manually count the number of branches and draw curves to obtain diameter lengths. These operations depended more or less on subjective vasculature definitions. However, our new method extracted LSAs in an automated way eliminating subjective influence. The reproducibility testing gave robust and consistent results, owing to its fixed and deterministic behavior. Therefore, this novel method would be suitable for perforating artery analyses in studies with larger populations.

According to previous studies, a consensus has been reached that LSA imaging has important clinical implications and provides insights into the mechanisms underlying the development of cerebral small vessel disease. However, radiologic studies investigating the associations between vasculature and basal ganglia are still lacking. Huge morphologic differences in LSA appearances among different individuals could be an important reason for the current limits of research on this topic, since currently available LSA imaging characteristics have poor sensitivity in being able to detect LSA abnormalities. In this study, the proposed method identified LSA vasculature and its supplying territories automatically. We showed a relationship between perforating arteries and the basal ganglia. The quantification of LSA vascular volume in subcortical areas could be used in both neurovascular studies and clinical diagnostic settings. LSA vascular volume reflects the density of perforating arteries and can be used to evaluate the impacts of lacunar infarcts, explore the causes of vascular dementia, and facilitate the etiologic studies of cerebral small vessel disease.

There are two major limitations to our study. First, large arteries were observed in the subcortical ROIs in 2 of 40 subjects, which interfered with the screening of LSA voxels and were thus removed from the analysis. These large vessels arose from the second MCA segments and crept along the surface of the brains without entering the parenchyma. Inclusion of these large arteries could be explained by misregistering subcortical ROIs from the templates to the individual spaces and should be improved upon in future studies. Second, when analyzing data from different subcortical areas, most vascular volume was found in the putamen, while very few voxels existed in other nuclei, especially the caudate nucleus. However, anatomic studies showed that LSAs arc around and course through the putamen and then travel superomedially through the superior part of the internal capsule and much of the caudate nucleus. The caudate nucleus is located superior to the putamen and other subcortical nuclei. The weakened in-flow of blood could have resulted in limited TOF-MRA sensitivity in the superior part of the scanning slab, and thus, miss small vessels in the caudate nucleus. However, the bias should not affect the results when comparing vascular volume in similar areas among different individuals.

## Conclusion

In conclusion, we proposed an automatic processing pipeline for quantifying LSA vascular volume in subcortical regions. The results provided a general knowledge of the relationship between perforating arteries and basal ganglia. This method introduces a new dimension for interpreting LSA imaging findings, and could potentially assist in the radiologic evaluation of neurodegenerative diseases caused by small vascular lesions.

## Data Availability Statement

The raw data supporting the conclusions of this article will be made available by the authors, without undue reservation.

## Ethics Statement

The studies involving human participants were reviewed and approved by The Institutional Review Board of Beijing MRI Center for Brain Research. The patients/participants provided their written informed consent to participate in this study.

## Author Contributions

NW and ZZ contributed to the conception and design of the study, analysis and interpretation of the data, and drafting of the manuscript. XZ and JA contributed to the acquisition of data. YZ contributed to the funding acquisition and supervision. All the authors approved the publication of the study.

## Conflict of Interest

XZ is an employee of Siemens Healthcare and JA is an employee of Siemens Shenzhen Magnetic Resonance Ltd. The Institute of Biophysics (Chinese Academy of Sciences) holds a research agreement with Siemens Healthcare. The remaining authors declare that the research was conducted in the absence of any commercial or financial relationships that could be construed as a potential conflict of interest.

## Publisher's Note

All claims expressed in this article are solely those of the authors and do not necessarily represent those of their affiliated organizations, or those of the publisher, the editors and the reviewers. Any product that may be evaluated in this article, or claim that may be made by its manufacturer, is not guaranteed or endorsed by the publisher.
